# Exploitation of Drought Tolerance-Related Genes for Crop Improvement

**DOI:** 10.3390/ijms221910265

**Published:** 2021-09-24

**Authors:** Jingyi Wang, Chaonan Li, Long Li, Matthew Reynolds, Xinguo Mao, Ruilian Jing

**Affiliations:** 1 National Key Facility for Crop Gene Resources and Genetic Improvement, Institute of Crop Sciences, Chinese Academy of Agricultural Sciences, Beijing 100081, China; wangjingyi@caas.cn (J.W.); lichaonan@caas.cn (C.L.); lilong01@caas.cn (L.L.); 2 International Maize and Wheat Improvement Center, Texcoco 56237, Mexico; M.REYNOLDS@CGIAR.ORG

**Keywords:** drought tolerance, phenotypic criteria, gene mining, genetic improvement, molecular breeding, crop plants

## Abstract

Drought has become a major threat to food security, because it affects crop growth and development. Drought tolerance is an important quantitative trait, which is regulated by hundreds of genes in crop plants. In recent decades, scientists have made considerable progress to uncover the genetic and molecular mechanisms of drought tolerance, especially in model plants. This review summarizes the evaluation criteria for drought tolerance, methods for gene mining, characterization of genes related to drought tolerance, and explores the approaches to enhance crop drought tolerance. Collectively, this review illustrates the application prospect of these genes in improving the drought tolerance breeding of crop plants.

## 1. Introduction

With population growth and climate change, food security has become a major global challenge. It is predicted that the rising demand will require a two-fold increase of food production by 2050 [1]. Crop production is being impacted by increasing abiotic stresses. Drought is one of the most severe abiotic stresses on crop production, and its impact depends on its timing, duration, and intensity, caused by insufficient rainfall and/or altered precipitation patterns. According to the Intergovernmental Panel on Climate Change reports, the annual area of dry lands during 1961–2013 has increased at a rate of average of more than 1% per year. In 2015, around 500 million people experienced desertification [2]. Future droughts are predicted to be more frequent, severe, and longer-lasting [3]. Therefore, it is urgent to accelerate the genetic improvement of crop drought tolerance by using desirable genes through the application of new biotechnological tools.

In the history of crop breeding, conventional techniques such as cross, backcross, artificial mutagenesis, pedigree selection, and recurrent selection have played an important role in the genetic improvement of drought tolerance. Conventional breeding mainly relies on phenotypic selection in the field; however, it is greatly affected by the environmental conditions and requires many years of identification and evaluation, resulting in highly labor-intensive, time-consuming, and low efficiency. To accelerate drought tolerance breeding, it is vital to understand the physiological and genetic basis of crop responses to drought. However, drought tolerance is a complex quantitative trait controlled by multiple genetic loci and susceptible to environmental influences. Therefore, in the present review, the evaluation criteria of crop drought tolerance were reviewed, followed by mining approaches of drought tolerance genes and methods to improve the drought tolerance of crops.

## 2. Crop Drought Tolerance Evaluation Criteria

The drought tolerance evaluation criteria are dependent on the timing, duration, and intensity of drought stress, climatic conditions, measurement time, location, and instruments. It is the first step of crop drought tolerance breeding to select appropriate criteria to evaluate the drought tolerance of crops in a specific period and within a specific environment. The following selection criteria have been used to distinguish drought tolerance genotypes.

### 2.1. Agronomic Criteria

#### 2.1.1. Morphological Traits

The visible phenotypes of plants can be used as an indicator of crop drought tolerance. For example, the rate and speed of seed germination, length of coleoptile, speed of leaf emergence, plant height, panicle neck diameter, wax content, survival rate under extreme drought, days to heading, seed setting rate, normalized difference vegetation index (NDVI), plant biomass, and their variations under different water regimes have been associated with drought tolerance [4].

Besides the above morphological traits, leaf morphology, including leaf length, width, thickness and color, leaf distribution, and stomatal characteristics, is highly responsive to a water deficit. Leaf rolling is also a typical physiological phenomenon under drought stress. Crop plants with moderately rolled leaves are thought to reduce water loss by transpiration [5]. Drought-tolerant plants tend to have some common phenotypes, such as smaller and thicker leaves, smaller and denser stomata, more epidermal trichomes, thicker cuticle epidermis and palisade tissue, and well-developed vascular bundle sheath.

Root architecture traits, such as the root number, diameter, angle, depth, total length, distribution, and biomass, are also highly associated with drought tolerance. Despite the importance of root traits in crop drought tolerance, they are rarely used directly as the selection criteria in breeding, because the phenotypic detection of root-related traits is a time-consuming and laborious process. Nonetheless, the root depth shows a strong association with a cooler canopy temperature, which can be used as a proxy in selection [6].

#### 2.1.2. Yield-Related Traits

Yield-related traits such as the yield index (YI), yield stability index (YSI), harvest index (HI), water use (WU), and yield based water use efficiency (WUE) were used to evaluate the performances of genotypes under drought stress and limited irrigation conditions [7]. 

### 2.2. Physiological and Biochemical Criteria

Plants have evolved a series of mechanisms to withstand drought stress not only at the morphological level but, also, at the physiological and biochemical levels. The physiological and biochemical characteristics of plants in response to water stress include the capacities of photosynthesis, osmotic adjustment, antioxidant defense, the hormone level, and enzyme activity [8].

#### 2.2.1. Photosynthesis-Related Traits

Drought severely decreases CO_2_ assimilation and affects photosynthesis by reducing the stomatal aperture and the concentration and activities of photosynthetic enzymes. Chlorophyll fluorescence is a relatively high throughput parameter to indicate photoinhibition due to a water deficit [9]. Fluorescence parameters were used to detect the changes of CO_2_ assimilation, linear electron flux, and photosystem II photochemistry under drought stress. Fluorescence imaging can be used to screen a large number of plants with photosynthetic perturbation under drought conditions. Crops with higher chlorophyll contents have a more efficient utilization of light energy and stronger drought tolerance under drought stress [10].

#### 2.2.2. Osmotic Adjustment-Related Traits

Since plants accumulate a variety of substances to maintain cell growth and leaf turgor, the concentration of these substances can reflect the crop drought tolerance to a certain extent. These substances can be classified into two catalogs: (1) inorganic ions, such as K^+^ and Cl^−^; (2) organic substances, such as trehalose, fructan, mannitol, proline, glycine, and betaine; and low molecular weight proteins such as late embryogenesis abundant (LEA) proteins, aquaporins (AQP), osmotin, and molecular chaperones.

#### 2.2.3. Antioxidant Defense-Related Traits

Oxidative stress is commonly accompanied by drought stress. Reactive oxygen species (ROS) stress may disturb the membrane protein and enzyme configuration [11]. The protective enzymes, such as superoxide dismutase (SOD), catalase (CAT), and peroxiredoxin (POD), are involved in reducing the ROS. Therefore, crop drought tolerance largely depends on the concentration of ROS and the activities of these protective enzymes.

#### 2.2.4. Phytohormones-Related Traits

Among the endogenous phytohormones, abscisic acid (ABA) is regarded as the most closely related plant hormone to drought stress response. Drought stress induces the biosynthesis and accumulation of intracellular ABA, mainly in the root cap and wilted leaves, which activates the corresponding transcription factors and then promotes the expression of downstream drought-related genes [12].

### 2.3. Integrated Drought Tolerance Criteria

Predicting crop drought tolerance based on the expression of morphological traits and/or physiological and biochemical characters is an essential step in breeding drought-tolerant crops. However, it is not easy to accurately evaluate the crop drought tolerance due to its complexity. Research has focused on investigating the efficiency of several criteria and integrating these criteria in identifying crop genotypes, combining the drought tolerance and high yield potential under stressed and nonstressed environments. Sahar et al. compared 24 indices and found strong positive correlations between the grain yield and nine indices, such as the mean productivity (MP), geometric mean productivity (GMP), stress tolerance index (STI), mean relative performance (MRP), relative efficiency index (REI), modified stress tolerance indices 1 and 2 (MSTIk), harmonic mean of yield (HM), and relative decrease in yield (RDY), which can be used for selecting drought tolerant and high-yield genotypes [13]. Khalili et al. proposed an integrated selection criterion index (SI) similar to MP, GMP, STI, stress susceptibility (SSI), and YSI as an effective selection criterion to distinguish the tolerant and desirable genotypes across multiple environments [14].

### 2.4. High-Throughput Phenotyping Platform

The emergence of a high-throughput phenotyping (HTP) platform makes it possible to capture trait information from a large number of plant samples under different water regimes [15]. The HTP platform integrated multiple imaging techniques, including red–green–blue (RGB), thermal infrared (TIR), chlorophyll fluorescence (ChlF), and multispectral and hyperspectral imaging and other sensor equipment [16]. Besides ground-based imaging, aerial imaging by unmanned aerial vehicles is also used to monitor the responses of crops to drought [17]. HTP technology provides a strong support for dissecting the genetic basis of drought tolerance in crops.

## 3. Methods for Mining Genes Related to Drought Tolerance

There are lots of omics approaches for mining genes related to drought tolerance in crop plants, such as genomics, transcriptomics, proteomics, metabolomics, ionomics, and epigenomics.

### 3.1. Genomics Analysis

With the development of high-throughput sequencing technology, the whole genome sequences of many crops have been unveiled (plaBiPD. Available online: http://www.plabipd.de/index.ep (accessed on 12 September 2021)). The availability of genomics platforms has made it easier to identify genes related to drought tolerance in crops. Nowadays, several genomics tools have been applied to elucidate drought tolerance-related genes, such as quantitative trait loci (QTL) mapping, map-based cloning, ectopic expression or suppression, genome-wide association studies (GWAS), and candidate gene association studies (CGAS).

#### 3.1.1. QTL Mapping

A number of QTL/genes for drought tolerant traits have been mapped, such as maize leaf temperature [18], maize leaf rolling index [19], rapeseed growth-related phenotypes [20], rice leaf rolling index [21], rice root angle [22], wheat stay-green trait under diverse water regimes [23], wheat stomatal conductance [24], density and size [25], and wheat yield-related phenotypes [26].

#### 3.1.2. Map-Based Cloning of Mutant

A major advantage of the map-based cloning approach is based on mutant phenotypes, and a series of genes involved in the mutant phenotypes can be discovered without prior assumptions or knowledge of these genes. With the improvement of crop reference genomic information, the availability of molecular markers, and the establishment of crop transformation systems, map-based cloning has become a conventional technique for gene discovery. Some genes related to drought tolerance were cloned, such as *PHOTO-SENSITIVE LEAF ROLLING 1* [5], *LEAF WILTING 3* [27], and the ABA receptor gene [28].

#### 3.1.3. Ectopic Expression or Suppression

The ectopic expression or suppression of target genes in different tissues or species is also an effective approach to identifying drought-tolerant genes. Through screening a wheat cDNA yeast library, wheat stress-responding genes were identified [29]. In addition, the overexpression, downregulation, or tissue-specific expression of artificially modified promoters is also an effective way to study gene functions [30]. For example, the drought response functions of *dehydration responsive element-binding protein*/*C-repeat-binding factor* (*DREB*/*CBF*) family genes, *GmDREB1* [31], *ZmDREB4.1* [32], *OsDREB2* [33], and *TaDREB3* [34] were identified by this approach. 

#### 3.1.4. GWAS and CGAS

With the development of high-density SNP detection techniques such as a DNA chip, high-throughput sequencing and genome-wide association studies on drought tolerance traits in many plant species have been widely carried out, such as soybean canopy wilting [35], maize survival rate [36], and wheat root depth [37], and a series of genes or loci were discovered. A candidate gene association study is based on the sequence polymorphism of genes, and through the analysis of the correlation between candidate genes and phenotypes, the favorable alleles are finally identified [38].

### 3.2. Transcriptomics Analysis

A transcriptomics analysis has been used to decipher drought tolerance-related genes such as *OsbZIP6* [39], the *LATERAL ROOT DENSITY* gene discovered from root transcriptome data [40]. Combining the transcriptomics data with the Kyoto Encyclopedia of Genes and Genomes (KEGG) and Gene Ontology (GO) enrichment analysis, the genes involved in some specific pathways could be predicted [41]. The expression of QTL (eQTL) and expression of GWAS (eGWAS) were also used to dissect the genetic basis of the drought-tolerant phenotype [42].

### 3.3. Proteomics Analysis

Drought stress has profound impacts on the protein abundance (such as protective proteins, chaperones, and ROS scavenging enzymes); post-translational modifications; protein interactions; and the ultimate functions of the proteins [43]. The comparison of protein profiling before and after drought stress or in genotypes with contrasting responses to drought stress will enhance our understanding of drought tolerance at the protein level [44].

#### 3.3.1. Mass Spectrometry

A series of techniques based on mass spectrometry (MS) are used for the proteomic analysis, such as gas chromatography MS (GC-MS), liquid chromatography MS (LC-MS), electrospray ionization (ESI), matrix-assisted laser desorption ionization with a time-of-flight analyzer (MALDI-TOF), and isobaric tags for relative and absolute quantification (iTRAQ) [45]. Using these techniques, not only the protein type and abundance can be detected but, also, protein modifications such as phosphorylation and ubiquitination can be analyzed.

#### 3.3.2. Two-Dimensional Gel Electrophoresis

A large number of proteomic data, including rice, tobacco, and Arabidopsis, were obtained using the techniques of two-dimensional gel electrophoresis and two-dimensional difference gel electrophoresis and are available at WORLD-2D-PAGE (Available online: https://world-2dpage.expasy.org/list/ (accessed on 12 September 2021)) [46].

#### 3.3.3. Protein Interaction Technique

Yeast two-hybrid (Y2H), yeast three-hybrid (Y3H), bimolecular fluorescence complementation (BiFC), luciferase complementation imaging assay (LCI), coimmunoprecipitation (CoIP), pull-down, and other protein interaction detection techniques can be used to detect drought tolerance-related proteins. For example, the ABA receptor was identified through the yeast two-hybrid screening of ABA-insensitive interaction proteins [47].

### 3.4. Metabolomics and Ionomics Analysis

Metabolomics is the study of small molecule profile based on the MS and nuclear magnetic resonance (NMR), including metabolites of small molecule substrates, intermediates, and products, which can be a prediction tool for plants’ performances under stress [48]. A better understanding of the metabolic response mechanisms under drought stress using metabolic QTL and metabolic GWAS could be beneficial to crop improvements [49].

Ionomics is a high-throughput elemental profiling method for studying the complete set of ions in living organisms. Therefore, it is widely used in forward [50] and reverse [51] genetics for detecting natural variants [52] and mutant screening to understand the mechanisms of abiotic stress tolerance in crop plants.

### 3.5. Epigenomics Analysis

Epigenetics studies epigenetic modifications at the genomic level, such as DNA methylation [53], histone modification [54], chromatin remodeling [55], and noncoding RNA [56]. Under environment stress, plants can transiently or lastingly change epigenetic modifications to adjust a gene expression to adapt to the environment [57]. For example, under drought stress, maize *natural antisense transcript* (*NAT*) genes are induced, and their gene regions are enriched with high levels of histone modifications, such as H3K36me3, H3K9ac, and H3K4me3 [58]. Under continuous stress, the identification of epigenetic markers that are heritable and transmitted to their progeny is of primary importance [59]. The role of DNA methylation variations in rice adaptations to drought stress was discovered by applying drought conditions to two rice varieties for 11 successive generations [60]. Plant microRNAs (miRNAs) also play a key role in the epigenetic regulation of drought tolerance [61]. Therefore, epigenomics study will also enhance our understanding of plant drought tolerance.

In conclusion, the comprehensive utilization of multi-omics technologies will facilitate mining drought tolerance-related genes for improving crop productivity under abiotic stress.

## 4. Genes Related to Drought Tolerance

A number of genes involved in drought tolerance have been discovered, and they fall into two main categories. One is signal transduction factors, including protein kinases and transcription factors. Another is functional factors, including proteins involved in metabolism, osmotic regulation, protein turnover, protein modification, and ROS scavenging and transportation. The detailed information is listed in Table 1.

### 4.1. Signal Transduction Factors

#### 4.1.1. ABA Receptors

Pyrabactin resistance 1/PYR1-like/regulatory components of the ABA receptor (PYR1/PYL/RCAR), as ABA receptors that were discovered through two methods: map-based cloning and Y2H at the same time, can perceive drought stress and transduce ABA signals [28,47]. In rice, the overexpression of *PYL9* [62] conferred drought tolerance. The overexpression of *TaPYL4* increased the water use efficiency and drought tolerance in wheat [63].

#### 4.1.2. Protein Kinases and Protein Phosphatases

The phosphorylation and dephosphorylation of proteins in plants play an important role in response to drought stress. A series of kinases were discovered to be participating in this process, such as calcium-dependent protein kinases (CDPKs), calcineurin B-like interacting protein kinase (CIPK), mitogen-activated protein kinases (MAPKs), receptor-like kinases (RLKs), and sucrose nonfermenting-related kinase 2s (SnRK2s). For example, (1) in CDPKs, GmCDPK3 was shown to positively regulate drought tolerance in soybeans [64]; (2) in CIPKs, CIPK genes have been known to be involved in abiotic stress tolerance in rice [65]; (3) in MAPKs, MPKK10.2 promoted the drought tolerance in rice [66]; (4) in RLKs, the RLK family member GbRLK from *Gossypium barbadense* enhanced the drought tolerance [67]; and (5) in SnRK2s, the SnRK2 family member TaSnRK2.9 conferred drought tolerance to transgenic tobacco [68]. Moreover, PP2C-type protein phosphatases are also highly associated with drought tolerance in both pathways: ABA-dependent, such as ZmPP2C-A [69], and ABA-independent signal transduction, such as OsPP2C09 [70].

#### 4.1.3. Transcription Factors and Cofactors

Diverse transcription factors such as AP2/ERF, bZIP, MYB, NAC, WRKY, and zinc finger protein have been known to be involved in the response to drought stress. The AP2/ERF transcription factor NtERF172 enhanced the drought tolerance through regulating NtCAT [71]. The ABA-responsive element-binding proteins/factors (AREBs/ABFs), which belong to the bZIP transcription factor, were involved in ABA-dependent drought response in wheat [12] and soybeans [72]. OsMYB102 negatively regulated ABA biosynthesis and the expression of downstream genes responding to abiotic stress [73]. Drought tolerance was enhanced in plants overexpressing the NAC family members *ZmNAC48* [74] and *TaNAC69* [75]. A zinc finger protein DST regulated the drought tolerance of rice by regulating the stomatal aperture [76].

A few nuclear proteins have been discovered as transcriptional cofactors in the drought response. MED25 subunit, which interacts with several stress response transcription factors as a RNA polymerase II coactivator and was involved in stress tolerance [77]. A rice homolog of the human ski-interacting protein OsSKIPa positively regulated the stress tolerance [78]. An overexpression of *OsRIP18*, rice ribosome-inactivating protein gene 18, increased the drought tolerance [79].

#### 4.1.4. Epigenetic-Related Genes

In rice, drought can induce ~12.1% site-specific DNA methylation differences at the genome level [80]. Increasing evidence indicates that epigenetic regulators, especially histone deacetylases, HDA9 [81] and HDA15 [82], and chromatin remodeling ATPase BRAHMA [83] were involved in the drought stress responsiveness. A number of drought-induced microRNAs have been identified in plants [84].

### 4.2. Functional Factors

#### 4.2.1. Metabolism-Related Genes

Plenty of metabolic-related proteins have been identified to participate in drought responses. The enzymes involved in ABA metabolism are one example. ABA plays an important role in drought tolerance by triggering stomatal closure to reduce the water loss. Zeaxanthin epoxidase (ZEP), 9-cis-epoxycarotenoid dioxygenase (NCED), and ABA-aldehyde oxidase (AAO) are involved in ABA biosynthesis. Plants overexpressing these genes enhance the drought tolerance [85,86]. In addition to these, LOS5/ABA3, a molybdenum cofactor sulfurase of AAO, increased the ABA accumulation and improved the drought tolerance in cotton [87] and soybeans [88]. The *DSM2* gene encoded the enzyme β-carotene hydroxylase, which converts β-carotene into zeaxanthin (a precursor of ABA) and can improve the drought tolerance of rice [89]. There are also genes (cytokinin biosynthetic gene *IPT*) involved in the metabolism of other hormones that affect the drought tolerance in cotton [90], rice [91], and tobacco [92].

The components involved in mRNA metabolism, including alternative splicing, RNA export, and pre-mRNA processing, play important roles in drought response. For example, the overexpression of *MeSCL30*, a cassava alternative splicing-related gene, enhanced the drought tolerance through maintaining ROS homeostasis and inducing drought-responsive gene expression [93]. Highly ABA-Induced 1 Interacting protein HIN1 with a pre-mRNA splicing function enhanced the splicing efficiency during drought acclimation [94]. Cold shock protein (CSP), functioning as an RNA chaperone, also participated in drought tolerance in Arabidopsis and maize [95].

Some genes involved in other substance metabolisms also contributed to the drought tolerance in crop plants. For example, arginine decarboxylase (ADC) modulated the polyamine biosynthetic pathway conferred drought tolerance of transgenic rice [96]. The overexpression of aspartic protease gene *APA1* also conferred a drought tolerance [97]. A C-5 sterol desaturase gene *FvC5SD* overexpression enhanced the drought tolerance in soybeans by reactive oxygen species scavenging [98]. A Caffeoyl-CoA O-methyltransferase gene conferred drought tolerance by promoting lignin synthesis [99]. The expression of flavodiiron proteins/reductases genes *Flv2*, *Flv3*, and *Flv4* in Arabidopsis and tobacco plants enhanced the drought tolerance [100].

#### 4.2.2. Osmotic Adjustment-Related Genes

Genes related to the synthesis and accumulation of osmotic adjustment substances can improve the drought tolerance of plants. For example, (1) dehydrin, an overexpression of the dehydrin gene *SiDHN*, promoted the cold and drought tolerance of transgenic tomato plants [101]; (2) glycine betaine, an overexpression of the betaine aldehyde dehydrogenase gene *BADH* from spinach, enhanced the drought and salinity tolerance of potato plants [102], and the choline oxidase gene *codA* converts choline into glycinebetaine, which was also associated with plant drought tolerance [103]; (3) LEA proteins, in which *LEA* genes were proven to play important roles in rice drought tolerance [104]; (4) mannitol, where an overexpression of the mannitol dehydrogenase gene *mtlD* imparted drought tolerance in finger millet [105]; (5) proline, where P5CS is a rate-limiting enzyme in proline biosynthesis, and its transformation increased proline production and enhanced the tolerance to water and salt stresses in rice [106]; an overexpression of the ornithine δ-aminotransferase gene *δ-OAT* increased the proline concentration and improved the drought tolerance [107]; and (6) trehalose, where trehalose-6-phosphate synthase (TPS) and trehalose-6-phosphate phosphatase (TPP) are two main enzymes in trehalose biosynthesis, and the overexpression of genes *TPS*/*TPP* increased the trehalose accumulation and drought tolerance in plants [108].

#### 4.2.3. Protein Turnover-Related Genes

Protein degradation is also a way for crop plants to adapt to drought stress. For example, the E3 ubiquitin ligase gene is involved in protein degradation during drought stress, which includes several subfamilies: (1) RING finger gene family members *OsDSG1* [109], *OsRDCP1* [110], *OsSDIR1* [111], *StRFP2* [112], and *TaSDIR1* [113]; (2) U-box gene family member *PUB11* [114]; (3) F-box gene family members *SAGL1* and *ECERIFERUM3* [115]; and (4) CUL4-DDB1 gene family member *HOS15* [116].

#### 4.2.4. Protein Modification-Related Genes

The genes involved in protein glycosylation, sumoylation, and farnesylation are also involved in response to drought stress. For example, *LEW3* [27] was involved in protein glycosylation and the abiotic stress response. SUMO protease *ASP1* [117] and SUMO E3 ligase *SIZ1* [118] had functions in ABA signaling. Protein farnesyltransferase was proven as a key negative ABA signal regulator in the guard cells of plants [119].

#### 4.2.5. ROS-Related Genes

Plant ROS scavenging has been demonstrated as an effective way to improve drought tolerance. Some ROS homeostasis-related genes have been proven to have functions in drought tolerance. For example, *OsSRO1c* conferred a rice drought tolerance through regulating hydrogen peroxide [120]. A pea *superoxide dismutase SOD* gene enhanced the drought tolerance in rice [121]. *EcAPX1*, *ascorbate peroxidase* from *Eleusine coracana*, played an important role in response to drought stress [122].

#### 4.2.6. Transporter Related Genes

Maize vacuolar-type H^+^ pyrophosphatase gene *ZmVPP1* contributed to seedling drought tolerance [36]. Pyramiding *TsVP* (V-H^+^-PPase from *Thellungiella halophila*) and *BetA* (choline dehydrogenase from *Escherichia coli*) through co-transformation enhanced the drought tolerance in maize [123]. *AQP* improved the drought tolerance, such as *OsRWC3* [124] and *MaPIP1;1* [125]. An auxin efflux carrier such as *OsPIN3t* was also involved in drought tolerance [126].

#### 4.2.7. Wax Related Genes

Lipid transfer proteins are involved in the biosynthesis of protective hydrophobic layers such as cutin and suberin [127]. Other wax-related genes, such as *DWA1* [128] and *Glossy1* homologous genes [129] in rice and *Glossy13* in maize [130], were involved in wax accumulation and drought tolerance. 

**Table 1:** Genes related to drought tolerance in crop plants.

Functional Category	Protein Function	Gene Name	Species	Method	Reference
**Signal Transduction Factor**					
ABA receptor	PYR/PYL/ RCAR	*OsPYL9*, *TaPYL4*	*Oryza sativa*, *Triticum aestivum*	Ectopic expression, transcriptomics	[62,63]
Protein kinase and protein phosphatase	CDPK	*GmCDPK3*	*Glycine max*	Transcriptomics	[64]
	CIPK	*OsCIPK*	*Oryza sativa*	Transcriptomics	[65]
	MAPK	*OsMPKK10.2*	*Oryza sativa*	Ectopic expression	[66]
	RLK	*GbRLK*	*Gossypium barbadense*	Ectopic expression	[67]
	SnRK	*TaSnRK2.9*	*Triticum aestivum*	Ectopic expression	[68]
	PP2C	*OsPP2C09*, *ZmPP2C*-*A*	*Oryza sativa*, *Zea mays*	CGAS, ectopic expression, transcriptomics	[69,70]
Transcription factor and cofactor	AP2/ERF	*NtERF172*	*Nicotiana tabacum*	Yeast one-hybrid	[71]
	bZIP	*GmbZIP1*, *TaAREB3*	*Glycine max*, *Triticum aestivum*	Ectopic expression, transcriptomics	[12,72]
	HD-ZIP	*ZmOCL5*	*Zea mays*	QTL mapping	[18]
	MYB	*OsMYB102*	*Oryza sativa*	Ectopic expression	[73]
	NAC	*TaNAC69*, *ZmNAC48*	*Triticum aestivum*, *Zea mays*	CGAS, ectopic expression, transcriptomics	[74,75]
	Zinc finger	*OsDST*	*Oryza sativa*	Map-based cloning	[76]
	Transcription cofactor	*OsSKIPa*, *OsRIP18*	*Oryza sativa*	Ectopic expression	[78,79]
**Functional factor**					
Metabolism	Abscisic acid metabolism	*OsDSM2*, *PvNCED1*	*Oryza sativa*, *Phaseolus vulgaris*	Ectopic expression, map-based cloning	[86,89]
	mRNA turnover	*MeSCL30*	*Manihot esculenta*	Transcriptomics	[93]
	Other substance metabolism	*ADCs*	*Avena Sativa*	Ectopic expression	[131]
Osmotic regulation	Dehydrin	*SiDHN*	*Saussurea involucrata*	Ectopic expression	[101]
	Glycine betaine	*BADH*	*Spinacia oleracea*	Ectopic expression	[102]
	LEA	*OsLEA3*-*2*	*Oryza sativa*	Ectopic expression	[104]
	Mannitol	*mtlD*	*Eleusine coracana*	Ectopic expression	[105]
	Proline	*OsP5CS*, *OsOAT*	*Oryza sativa*	Ectopic expression	[106,107]
	Trehalose	*ZmT6P*	*Zea mays*	Ectopic expression	[108]
Protein turnover	E3 ubiquitin ligase	*StRFP2*, *TaSDIR1*	*Solanum tuberosm*, *Triticum aestivum*	CGAS, ectopic expression	[112,113]
Protein modification	Farnesylation	*ERA1*	*Brassica napus*	Ectopic expression	[119]
	Sumoylation	*OsSIZ1*	*Oryza sativa*	Ectopic expression	[118]
ROS scavenging	Ascorbate peroxidase	*EcAPX1*	*Eleusine coracana*	Ectopic expression	[122]
	Similar to RCD One	*OsSRO1c*	*Oryza sativa*	Ectopic expression	[120]
	Superoxide dismutase	*SOD*	*Pisum sativum*	Ectopic expression	[121]
Transporters	Aquaporin	*MaPIP1;1*, *OsRWC3*	*Musa acuminata*, *Oryza sativa*	Ectopic expression	[124,125]
	Auxin efflux carrier	*OsPIN3t*	*Oryza sativa*	Ectopic expression	[126]
	Choline Dehydrogenase, V-H^+^-PPase	*ZmbetA*, *ZmTsVP*	*Zea mays*	Ectopic expression	[123]
	Vacuolar-type H^+^ pyrophosphatase	*ZmVPP1*	*Zea mays*	GWAS	[36]
Cuticle wax	ABC transporter	*ZmGLOSSY13*	*Zea mays*	Map-based cloning	[130]
	Megaenzyme	*OsDWA1*	*Oryza sativa*	Map-based cloning	[128]
	Sterol desaturase	*OsGLOSSY1*	*Oryza sativa*	Ectopic expression	[129]

## 5. Genetic Improvement of Drought Tolerance in Crops

In the history of crop breeding, breeders have unconsciously selected target genes by evaluating phenotypic traits. However, conventional breeding, which relies on multilocation phenotypic selection for many years, is inefficient. The application of molecular breeding technology will facilitate the direct selection of genotypes, speed up the breeding process, and improve the efficiency of developing drought-tolerant varieties.

### 5.1. Marker-Assisted Selection

Marker-assisted selection (MAS) is a selection strategy based on QTL or gene markers employed by breeders to accelerate plant breeding programs. According to the purpose of selection, MAS can be divided into marker-assisted pedigree selection (MAPS), genomic selection (GS) or genome-wide selection (GWS), marker-assisted recurrent selection (MARS), and marker-assisted backcrossing (MABC). Gupta et al. [132] reviewed the wheat genetics of important quantitative traits, including a tolerance to abiotic stress, and summarized the potential value of QTLs for the improvement of drought tolerance using MAS. Despite the availability of a large number of major QTL for drought tolerance, little progress has been made for MAS. For example, in rice, the pyramiding of six large-effect QTL for drought adaptation has improved the drought tolerance of Asian cultivars [133]. In wheat, using MAS, a major QTL (Qyld.csdh.7AL) was introgressed into wheat cultivars to develop a high-yield genotype under rainfed conditions [134]; the introgression of QTL on 7AS and 2BS led to the improvement of the grain yield under the drought treatment [135]. Functional molecular markers of candidate genes for drought tolerance have also been developed for use in breeding [136].

### 5.2. Genomic Selection

MAS has been implemented for crop breeding, but its efficiency is limited due to the small number of molecular markers that can be used. Drought and most agronomic traits are quantitative traits controlled by multiple minor effect genes. Using genome-wide markers to predict the breeding values of individuals, genomic selection as a promising breeding method presents a new alternative to traditional MAS [137]. GS has been shown to improve the efficiency and speed up breeding in maize [138], rice [139], and wheat [140], and high prediction accuracies have been obtained for the yield and a number of other traits. However, due to the genetic complexity of its characteristics, the genomic selection for improving drought tolerance should be investigated.

### 5.3. Genetic Improvement Using Transgene and Genome Editing Techniques

Along with the identification of the candidate genes, we can also use transgenic technology to improve the crop drought tolerance, such as OE, RNAi, VIGS, ZFN, TALEN, and CRISPR [141]. At present, genetically modified crops are widely used in the world. The genes used in transgenic crops mainly include herbicide resistance gene (*Bar*), insect resistance gene (*Bt*), and disease resistance genes, while drought tolerance genes are rarely used. Transgenic technology and gene editing technology will promote the genetic improvement of crop drought resistance. For example, CRISPR technologies can not only introduce a small insertion or deletion mutations at the target loci but can also provide precision editing, such as base editing, prime editing, and gene targeting [142].

### 5.4. Genetic Improvement Combined with the Chemical Approach

As an important hormone, ABA plays a vital role in the drought tolerance of crops, while, due to the chemical instability, the rapid catabolism of ABA limits its application in the field. Scientists have conducted vast amounts of research to search for more stable ABA analogs, such as pyrabactin, AM1 (ABA mimic 1)/quinabactin, cyanabactin, opabactin, and AMFs [143]. Furthermore, combining chemical and genetic approaches, AMFs are applied to *PYL2* overexpression transgenic plants, increasing their drought tolerance [144]. Another avenue to improve crop drought tolerance is engineering an ABA receptor. An ABA receptor PYR1 variant, which is sensitive to the agrochemical mandipropamid, improved the drought tolerance in transgenic plants [145].

### 5.5. Molecular Design Breeding

Molecular design breeding, a highly integrated system based on biotechnology and bioinformatics [146], may be an effective approach to enhance drought tolerance [147]. It can design and manipulate genotypes to meet various breeding objectives in different ecological regions under diverse water conditions [148]. Based on the principle of molecular design breeding, the ideal genotypes can be identified during crossing, selection and transgene and genome editing.

## 6. Conclusions and Perspectives

Most transgenic plants with drought tolerance phenotypes were based on the overexpression of genes using constitutive promoters, such as actin, CaMV35S, and ubiquitin. However, the overexpression of genes is often accompanied by a yield penalty due to stress-induced energy consumption. Therefore, mining drought tolerance-related genes that are not subjected to yield penalties or fine-tuning these genes through suitable stress-inducible promoters to minimize the yield penalties should be considered [149].

Most of the genes reviewed in this paper have been shown to enhance drought tolerance in growth chambers or greenhouses, and the utility of most of the genes has not been verified in the field. This is especially problematic for water stress, since root growth in pots in controlled environments cannot be compared to their response in deep soil water profiles, which has a profound impact on their adaptation and productivity. Therefore, the use of these drought-tolerant genes in crop breeding still needs the joint efforts of scientists and breeders to achieve a proof of concept.

**Figure 1:**
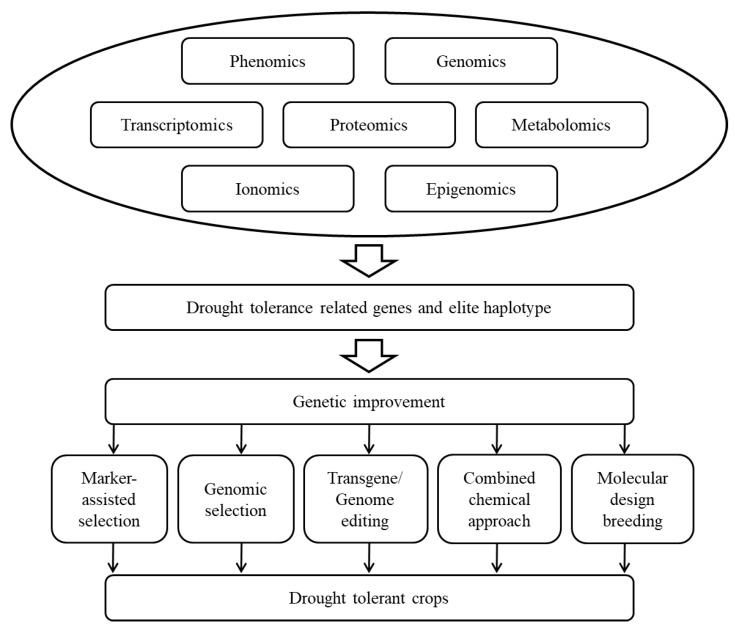
Approaches of breeding for drought tolerance in crops.

Crop plants are subjected to drought stress at various stages of growth and development. Plants can adapt to drought stress through a series of morphological, physiological, and biochemical changes, but these genetically regulated responses are extremely complex. Therefore, it is necessary to conduct phenotypic and genotypic identification and evaluation through multidisciplinary approaches and to comprehensively use conventional, physiological, and molecular breeding techniques to improve the drought tolerance of crops (Figure 1).

## Data Availability

Not applicable.

## Abbreviations

AAO	ABA-aldehyde oxidase
ABA	Abscisic acid
AQP	Aquaporins
AREB/ABF	ABA-responsive element-binding proteins/factors
CDPK	Calcium dependent protein kinase
CGAS	Candidate gene association study
CIPK	Calcineurin B-like interacting protein kinase
CSP	Cold shock protein
DREB/CBF	Dehydration responsive element binding protein/C-repeat binding factor
GWAS	Genome wide association studies
HTP	High throughput phenotyping
LEA	Late embryogenesis abundant
MAPK	Mitogen-activated protein kinase
MAS	Marker-assisted selection
PYR1/PYL/RCAR	Pyrabactin resistance 1/PYR1-like/Regulatory components of the ABA receptor
QTL	Quantitative trait loci
RLK	Receptor like kinase
ROS	Reactive oxygen species
SnRK2	Sucrose nonfermenting related kinase 2
SOD	Superoxide dismutase
TPS	Trehalose-6-phosphate synthase

## References

[1] Henry R.J. (2020). Innovations in plant genetics adapting agriculture to climate change. Curr. Opin. Plant Biol..

[2] Shukla P.R., Skea J., Calvo Buendia E., Masson-Delmotte V., Pörtner H.-O., Roberts D.C., Zhai P.M., Slade R., Connors S., Diemen R. (2020). IPCC, 2019: Summary for Policymakers. Climate Change and Land: An IPCC Special Report on Climate Change, Desertification, Land Degradation, Sustainable Land Management, Food Security, and Greenhouse Gas Fluxes in Terrestrial Ecosystems.

[3] Ault T.R. (2020). On the essentials of drought in a changing climate. Science.

[4] Li L., Mao X.G., Wang J.Y., Chang X.P., Reynolds M., Jing R.L. (2019). Genetic dissection of drought and heat-responsive agronomic traits in wheat. Plant Cell Environ..

[5] Zhang G.H., Hou X., Wang L., Xu J., Chen J., Fu X., Shen N.W., Nian J.Q., Jiang Z.Z., Hu J. (2021). Photo-sensitive leaf rolling 1 encodes a polygalacturonase that modifies cell wall structure and drought tolerance in rice. New Phytol..

[6] Lopes M.S., Reynolds M.P. (2010). Partitioning of assimilates to deeper roots is associated with cooler canopies and increased yield under drought in wheat. Funct. Plant Biol.

[7] Salekdeh G.H., Reynolds M., Bennett J., Boyer J. (2009). Conceptual framework for drought phenotyping during molecular breeding. Trends Plant Sci..

[8] Sreeman S.M., Vijayaraghavareddy P., Sreevathsa R., Rajendrareddy S., Arakesh S., Bharti P., Dharmappa P., Soolanayakanahally R. (2018). Introgression of physiological traits for a comprehensive improvement of drought adaptation in crop plants. Front. Chem..

[9] Ashraf M., Harris P.J.C. (2013). Photosynthesis under stressful environments: An overview. Photosynthetica.

[10] Shin Y.K., Bhandari S.R., Cho M.C., Lee J.G. (2020). Evaluation of chlorophyll fluorescence parameters and proline content in tomato seedlings grown under different salt stress conditions. Hortic. Environ. Biotechnol..

[11] Gill S.S., Tuteja N. (2010). Reactive oxygen species and antioxidant machinery in abiotic stress tolerance in crop plants. Plant Physiol. Biochem..

[12] Wang J.Y., Li Q., Mao X.G., Li A., Jing R.L. (2016). Wheat transcription factor TaAREB3 participates in drought and freezing tolerances in *Arabidopsis*. Int. J. Biol. Sci..

[13] Bennani S., Birouk A., Nsarellah N., Jlibene M., Ouabbou H. (2016). Efficiency of selection indices in screening bread wheat lines combining drought tolerance and high yield potential. J. Plant Breed. Crop. Sci..

[14] Khalili M., Pour-Aboughadareh A., Naghavi M.R. (2016). Assessment of drought tolerance in barley: Integrated selection criterion and drought tolerance indices. Environ. Exp. Biol..

[15] Junker A., Muraya M.M., Weigelt-Fischer K., Arana-Ceballos F., Klukas C., Melchinger A.E., Meyer R.C., Riewe D., Altmann T. (2015). Optimizing experimental procedures for quantitative evaluation of crop plant performance in high throughput phenotyping systems. Front. Plant Sci..

[16] Banan D., Paul R.E., Feldman M.J., Holmes M.W., Schlake H., Baxter I., Jiang H., Leakey A.D.B. (2018). High-fidelity detection of crop biomass quantitative trait loci from low-cost imaging in the field. Plant Direct..

[17] Baluja J., Diago M.P., Balda P., Zorer R., Meggio F., Morales F., Tardaguila J. (2012). Assessment of vineyard water status variability by thermal and multispectral imagery using an unmanned aerial vehicle (UAV). Irrigation Sci..

[18] Liu Y., Subhash C., Yan J.B., Song C.P., Zhao J.R., Li J.S. (2011). Maize leaf temperature responses to drought: Thermal imaging and quantitative trait loci (QTL) mapping. Environ. Exp. Bot..

[19] Gao L.L., Yang G.H., Li Y.F., Fan N.N., Li H.J., Zhang M., Xu R.B., Zhang M.Y., Zhao A.J., Ni Z.F. (2019). Fine mapping and candidate gene analysis of a QTL associated with leaf rolling index on chromosome 4 of maize (*Zea mays* L.). Theor. Appl. Genet..

[20] Fletcher R.S., Mullen J.L., Heiliger A., McKay J.K. (2015). QTL analysis of root morphology, flowering time, and yield reveals trade-offs in response to drought in *Brassica napus*. J. Exp. Bot..

[21] Zhang Q., Zheng T.Q., Hoang L., Wang C.C., Nafisah, Joseph C., Zhang W.Z., Xu J.L., Li Z.K. (2016). Joint mapping and allele mining of the rolled leaf trait in rice (*Oryza sativa* L.). PLoS ONE.

[22] Uga Y., Sugimoto K., Ogawa S., Rane J., Ishitani M., Hara N., Kitomi Y., Inukai Y., Ono K., Kanno N. (2013). Control of root system architecture by *DEEPER ROOTING 1* increases rice yield under drought conditions. Nat. Genet..

[23] Shi S.K., Azam F.I., Li H.H., Chang X.P., Li B.Y., Jing R.L. (2017). Mapping QTL for stay-green and agronomic traits in wheat under diverse water regimes. Euphytica.

[24] Wang S.G., Jia S.S., Sun D.Z., Wang H.Y., Dong F.F., Ma H.X., Jing R.L., Ma G. (2015). Genetic basis of traits related to stomatal conductance in wheat cultivars in response to drought stress. Photosynthetica.

[25] Wang S.G., Jia S.S., Sun D.Z., Fan H., Chang X.P., Jing R.L. (2016). Mapping QTLs for stomatal density and size under drought stress in wheat (*Triticum aestivum* L.). J. Integr. Agric..

[26] Wu X.S., Chang X.P., Jing R.L. (2012). Genetic insight into yield-associated traits of wheat grown in multiple rain-fed environments. PLoS ONE.

[27] Zhang M., Henquet M., Chen Z.Z., Zhang H.R., Zhang Y., Ren X.Z., van der Krol S., Gonneau M., Bosch D., Gong Z.Z. (2009). *LEW3*, encoding a putative alpha-1,2-mannosyltransferase (ALG11) in N-linked glycoprotein, plays vital roles in cell-wall biosynthesis and the abiotic stress response in *Arabidopsis thaliana*. Plant J..

[28] Park S.Y., Fung P., Nishimura N., Jensen D.R., Fujii H., Zhao Y., Lumba S., Santiago J., Rodrigues A., Chow T.F.F. (2009). Abscisic acid inhibits type 2c protein phosphatases via the PYR/PYL family of START proteins. Science.

[29] Wang J.Y., Mao X.G., Wang R.T., Li A., Zhao G.Y., Zhao J.F., Jing R.L. (2019). Identification of wheat stress-responding genes and *TaPR-1-1* function by screening a cDNA yeast library prepared following abiotic stress. Sci. Rep..

[30] Zhu Y.F., Wang B.S., Tang K., Hsu C.C., Xie S.J., Du H., Yang Y.T., Tao W.A., Zhu J.K. (2017). An Arabidopsis Nucleoporin NUP85 modulates plant responses to ABA and salt stress. PLoS Genet..

[31] Zhou Y.B., Chen M., Guo J.K., Wang Y.X., Min D.H., Jiang Q.Y., Ji H.T., Huang C.Y., Wei W., Xu H.J. (2020). Overexpression of soybean *DREB1* enhances drought stress tolerance of transgenic wheat in the field. J. Exp. Bot..

[32] Li S.X., Zhao Q., Zhu D.Y., Yu J.J. (2018). A DREB-like transcription factor from maize (*Zea mays*), ZmDREB4.1, plays a negative role in plant growth and development. Front. Plant Sci..

[33] Herath V. (2016). Small family, big impact: In silico analysis of DREB2 transcription factor family in rice. Comput. Biol. Chem..

[34] Niu X., Luo T.L., Zhao H.Y., Su Y.L., Ji W.Q., Li H.F. (2020). Identification of wheat *DREB* genes and functional characterization of *TaDREB3* in response to abiotic stresses. Gene.

[35] Steketee C.J., Schapaugh W.T., Carter T.E., Li Z.L. (2020). Genome-wide association analyses reveal genomic regions controlling canopy wilting in soybean. G3 Genes Genom. Genet..

[36] Wang X.L., Wang H.W., Liu S.X., Ferjani A., Li J.S., Yan J.B., Yang X.H., Qin F. (2016). Genetic variation in *ZmVPP1* contributes to drought tolerance in maize seedlings. Nat. Genet..

[37] Wang J.Y., Li L., Li C.N., Yang X., Xue Y.H., Zhu Z., Mao X.G., Jing R.L. (2021). A transposon in the vacuolar sorting receptor gene *TaVSR1-B* promoter region is associated with wheat root depth at booting stage. Plant Biotechnol. J..

[38] Guo H.L., Wang Y., Zhang B., Li D.D., Chen J.B., Zong J.Q., Li J.J., Liu J.X., Jiang Y.W. (2019). Association of candidate genes with drought tolerance traits in zoysiagrass germplasm. J. Plant Physiol..

[39] Yang S.Q., Xu K., Chen S.J., Li T.F., Xia H., Chen L., Liu H.Y., Luo L.J. (2019). A stress-responsive bZIP transcription factor *OsbZIP62* improves drought and oxidative tolerance in rice. BMC Plant Biol..

[40] Placido D.F., Sandhu J., Sato S.J., Nersesian N., Quach T., Clemente T.E., Staswick P.E., Walia H. (2020). The *LATERAL ROOT DENSITY* gene regulates root growth during water stress in wheat. Plant Biotechnol. J..

[41] Kanehisa M., Furumichi M., Tanabe M., Sato Y., Morishima K. (2017). KEGG: New perspectives on genomes, pathways, diseases and drugs. Nucleic Acids Res..

[42] Lowry D.B., Logan T.L., Santuari L., Hardtke C.S., Richards J.H., DeRose-Wilson L.J., McKay J.K., Sen S., Juenger T.E. (2013). Expression quantitative trait locus mapping across water availability environments reveals contrasting associations with genomic features in *Arabidopsis*. Plant Cell.

[43] Kosova K., Vitamvas P., Urban M.O., Prasil I.T., Renaut J. (2018). Plant abiotic stress proteomics: The major factors determining alterations in cellular proteome. Front. Plant Sci..

[44] Chaudhary S., Jabre I., Reddy A.S.N., Staiger D., Syed N.H. (2019). Perspective on alternative splicing and proteome complexity in plants. Trends Plant Sci..

[45] Freitas J.R.L., Vendramini P.H., Melo J.O.F., Eberlin M.N., Augusti R. (2018). An appraisal on the source-to-sink relationship in plants: An application of desorption electrospray ionization mass spectrometry imaging. J. Brazil Chem. Soc..

[46] Van den Bergh G., Arckens L. (2004). Fluorescent two-dimensional difference gel electrophoresis unveils the potential of gel-based proteomics. Curr. Opin. Biotechnol..

[47] Ma Y., Szostkiewicz I., Korte A., Moes D., Yang Y., Christmann A., Grill E. (2009). Regulators of PP2C phosphatase activity function as abscisic acid sensors. Science.

[48] Villate A., San Nicolas M., Gallastegi M., Aulas P.-A., Olivares M., Usobiaga A., Etxebarria N., Aizpurua-Olaizola O. (2021). Review: Metabolomics as a prediction tool for plants performance under environmental stress. Plant Sci..

[49] Francki M.G., Hayton S., Gummer J.P.A., Rawlinson C., Trengove R.D. (2016). Metabolomic profiling and genomic analysis of wheat aneuploid lines to identify genes controlling biochemical pathways in mature grain. Plant Biotechnol. J..

[50] Baxter I., Hosmani P.S., Rus A., Lahner B., Borevitz J.O., Muthukumar B., Mickelbart M.V., Schreiber L., Franke R.B., Salt D.E. (2009). Root suberin forms an extracellular barrier that affects water relations and mineral nutrition in *Arabidopsis*. PLoS Genet..

[51] Chao D.Y., Gable K., Chen M., Baxter I., Dietrich C.R., Cahoon E.B., Guerinot M.L., Lahner B., Lu S.Y., Markham J.E. (2011). Sphingolipids in the root play an important role in regulating the leaf ionome in *Arabidopsis thaliana*. Plant Cell.

[52] Chen Z., Watanabe T., Shinano T., Ezawa T., Wasaki J., Kimura K., Osaki M., Zhu Y.G. (2009). Element interconnections in *Lotus japonicus*: A systematic study of the effects of element additions on different natural variants. Soil. Sci. Plant Nutr..

[53] Li R.X., Hu F., Li B., Zhang Y.P., Chen M., Fan T., Wang T.C. (2020). Whole genome bisulfite sequencing methylome analysis of mulberry (*Morus alba*) reveals epigenome modifications in response to drought stress. Sci. Rep..

[54] Song J., Henry H., Tian L. (2020). Drought-inducible changes in the histone modification H3K9ac are associated with drought-responsive gene expression in *Brachypodium distachyon*. Plant Biol..

[55] Lamke J., Baurle I. (2017). Epigenetic and chromatin-based mechanisms in environmental stress adaptation and stress memory in plants. Genome Biol..

[56] Xu J., Hou Q.M., Khare T., Verma S.K., Kumar V. (2019). Exploring miRNAs for developing climate-resilient crops: A perspective review. Sci. Total Environ..

[57] Varotto S., Tani E., Abraham E., Krugman T., Kapazoglou A., Melzer R., Radanovic A., Miladinovic D. (2020). Epigenetics: Possible applications in climate-smart crop breeding. J. Exp. Bot..

[58] Xu J., Wang Q., Freeling M., Zhang X.C., Xu Y.B., Mao Y., Tang X., Wu F.K., Lan H., Cao M.J. (2017). Natural antisense transcripts are significantly involved in regulation of drought stress in maize. Nucleic Acids Res..

[59] Mladenov V., Fotopoulos V., Kaiserli E., Karalija E., Maury S., Baranek M., Segal N., Testillano P.S., Vassileva V., Pinto G. (2021). Deciphering the epigenetic alphabet involved in transgenerational stress memory in crops. Int. J. Mol. Sci..

[60] Zheng X.G., Chen L., Xia H., Wei H.B., Lou Q.J., Li M.S., Li T.M., Luo L.J. (2017). Transgenerational epimutations induced by multi-generation drought imposition mediate rice plant’s adaptation to drought condition. Sci. Rep..

[61] Nadarajah K., Kumar I.S. (2019). Drought response in rice: The miRNA story. Int. J. Mol. Sci..

[62] Zhao Y., Chan Z.L., Gao J.H., Xing L., Cao M.J., Yu C.M., Hu Y.L., You J., Shi H.T., Zhu Y.F. (2016). ABA receptor PYL9 promotes drought resistance and leaf senescence. Proc. Natl. Acad. Sci. USA.

[63] Mega R., Abe F., Kim J.S., Tsuboi Y., Tanaka K., Kobayashi H., Sakata Y., Hanada K., Tsujimoto H., Kikuchi J. (2019). Tuning water-use efficiency and drought tolerance in wheat using abscisic acid receptors. Nat. Plants.

[64] Wang D., Liu Y.X., Yu Q., Zhao S.P., Zhao J.Y., Ru J.N., Cao X.Y., Fang Z.W., Chen J., Zhou Y.B. (2019). Functional analysis of the soybean *GmCDPK3* gene responding to drought and salt stresses. Int. J. Mol. Sci..

[65] Xiang Y., Huang Y.M., Xiong L.Z. (2007). Characterization of stress-responsive *CIPK* genes in rice for stress tolerance improvement. Plant Physiol..

[66] Ma H.G., Chen J., Zhang Z.Z., Ma L., Yang Z.Y., Zhang Q.L., Li X.H., Xiao J.H., Wang S.P. (2017). MAPK kinase 10.2 promotes disease resistance and drought tolerance by activating different MAPKs in rice. Plant J..

[67] Zhao J., Gao Y.L., Zhang Z.Y., Chen T.Z., Guo W.Z., Zhang T.Z. (2013). A receptor-like kinase gene (*GbRLK*) from *Gossypium barbadense* enhances salinity and drought-stress tolerance in *Arabidopsis*. BMC Plant Biol..

[68] Feng J.L., Wang L.Z., Wu Y.N., Luo Q.C., Zhang Y., Qiu D., Han J.P., Su P.P., Xiong Z.Y., Chang J.L. (2019). *TaSnRK2.9*, a sucrose non-fermenting 1-related protein kinase gene, positively regulates plant response to drought and salt stress in transgenic tobacco. Front. Plant Sci..

[69] Xiang Y.L., Sun X.P., Gao S., Qin F., Dai M.Q. (2017). Deletion of an endoplasmic reticulum stress response element in a *ZmPP2C-A* gene facilitates drought tolerance of maize seedlings. Mol. Plant.

[70] Min M.K., Kim R., Hong W.J., Jung K.H., Lee J.Y., Kim B.G. (2021). OsPP2C09 is a bifunctional regulator in both ABA-dependent and independent abiotic stress signaling pathways. Int. J. Mol. Sci..

[71] Zhao Q., Hu R.S., Liu D., Liu X., Wang J., Xiang X.H., Li Y.Y. (2020). The AP2 transcription factor *NtERF172* confers drought resistance by modifying *NtCAT*. Plant Biotechnol. J..

[72] Xu S.M., Song S.S., Dong X.X., Wang X.Y., Wu J., Ren Z.Y., Wu X.S., Lu J.J., Yuan H.F., Wu X.Y. (2021). GmbZIP1 negatively regulates ABA-induced inhibition of nodulation by targeting *GmENOD40-1* in soybean. BMC Plant Biol..

[73] Piao W., Sakuraba Y., Paek N.C. (2019). Transgenic expression of rice *MYB102* (*OsMYB102*) delays leaf senescence and decreases abiotic stress tolerance in *Arabidopsis thaliana*. BMB Rep..

[74] Mao Y., Xu J., Wang Q., Li G.B., Tang X., Liu T.H., Feng X.J., Wu F.K., Li M.L., Xie W.B. (2021). A natural antisense transcript acts as a negative regulator for the maize drought stress response gene *ZmNAC48*. J. Exp. Biol..

[75] Xue G.P., Way H.M., Richardson T., Drenth J., Joyce P.A., McIntyre C.L. (2011). Overexpression of *TaNAC69* leads to enhanced transcript levels of stress up-regulated genes and dehydration tolerance in bread wheat. Mol. Plant.

[76] Huang X.Y., Chao D.Y., Gao J.P., Zhu M.Z., Shi M., Lin H.X. (2009). A previously unknown zinc finger protein, DST, regulates drought and salt tolerance in rice via stomatal aperture control. Gene Dev..

[77] Elfving N., Davoine C., Benlloch R., Blomberg J., Brannstrom K., Muller D., Nilsson A., Ulfstedt M., Ronne H., Wingsle G. (2011). The *Arabidopsis thaliana* Med25 mediator subunit integrates environmental cues to control plant development. Proc. Natl. Acad. Sci. USA.

[78] Hou X., Xie K.B., Yao J.L., Qi Z.Y., Xiong L.Z. (2009). A homolog of human ski-interacting protein in rice positively regulates cell viability and stress tolerance. Proc. Natl. Acad. Sci. USA.

[79] Jiang S.Y., Bhalla R., Ramamoorthy R., Luan H.F., Venkatesh P.N., Cai M.N., Ramachandran S. (2012). Over-expression of *OSRIP18* increases drought and salt tolerance in transgenic rice plants. Transgenic. Res..

[80] Wang W.S., Pan Y.J., Zhao X.Q., Dwivedi D., Zhu L.H., Ali J., Fu B.Y., Li Z.K. (2011). Drought-induced site-specific DNA methylation and its association with drought tolerance in rice (*Oryza sativa* L.). J. Exp. Bot..

[81] Zheng Y., Ding Y., Sun X., Xie S.S., Wang D., Liu X.Y., Su L.F., Wei W., Pan L., Zhou D.X. (2016). Histone deacetylase HDA9 negatively regulates salt and drought stress responsiveness in *Arabidopsis*. J. Exp. Bot..

[82] Lee H.G., Seo P.J. (2019). MYB96 recruits the HDA15 protein to suppress negative regulators of ABA signaling in *Arabidopsis*. Nat. Commun..

[83] Peirats-Llobet M., Han S.K., Gonzalez-Guzman M., Jeong C.W., Rodriguez L., Belda-Palazon B., Wagner D., Rodriguez P.L. (2016). A direct link between abscisic acid sensing and the chromatin-remodeling ATPase BRAHMA via core ABA signaling pathway components. Mol. Plant.

[84] Fracasso A., Vallino M., Staropoli A., Vinale F., Amaducci S., Carra A. (2021). Increased water use efficiency in miR396-downregulated tomato plants. Plant Sci..

[85] Park H.Y., Seok H.Y., Park B.K., Kim S.H., Goh C.H., Lee B., Lee C.H., Moon Y.H. (2008). Overexpression of *Arabidopsis ZEP* enhances tolerance to osmotic stress. Biochem. Biophys. Res. Commun..

[86] Qin X.Q., Zeevaart J.A.D. (2002). Overexpression of a 9-*cis*-epoxycarotenoid dioxygenase gene in *Nicotiana plumbaginifolia* increases abscisic acid and phaseic acid levels and enhances drought tolerance. Plant Physiol..

[87] Yue Y.S., Zhang M.C., Zhang J.C., Tian X.L., Duan L.S., Li Z.H. (2012). Overexpression of the *AtLOS5* gene increased abscisic acid level and drought tolerance in transgenic cotton. J. Exp. Bot..

[88] Li Y.J., Zhang J.C., Zhang J., Hao L., Hua J.P., Duan L.S., Zhang M.C., Li Z.H. (2013). Expression of an *Arabidopsis* molybdenum cofactor sulphurase gene in soybean enhances drought tolerance and increases yield under field conditions. Plant Biotechnol. J..

[89] Du H., Wang N.L., Cui F., Li X.H., Xiao J.H., Xiong L.Z. (2010). Characterization of the beta-carotene hydroxylase gene *DSM2* conferring drought and oxidative stress resistance by increasing xanthophylls and abscisic acid synthesis in rice. Plant Physiol..

[90] Kuppu S., Mishra N., Hu R.B., Sun L., Zhu X.L., Shen G.X., Blumwald E., Payton P., Zhang H. (2013). Water-deficit inducible expression of a cytokinin biosynthetic gene *IPT* improves drought tolerance in cotton. PLoS ONE.

[91] Peleg Z., Reguera M., Tumimbang E., Walia H., Blumwald E. (2011). Cytokinin-mediated source/sink modifications improve drought tolerance and increase grain yield in rice under water-stress. Plant Biotechnol. J..

[92] Rivero R.M., Kojima M., Gepstein A., Sakakibara H., Mittler R., Gepstein S., Blumwald E. (2007). Delayed leaf senescence induces extreme drought tolerance in a flowering plant. Proc. Natl. Acad. Sci. USA.

[93] Weng X., Zhou X.X., Xie S.Q., Gu J.B., Wang Z.Y. (2021). Identification of cassava alternative splicing-related genes and functional characterization of *MeSCL30* involvement in drought stress. Plant Physiol. Biochem..

[94] Chong G.L., Foo M.H., Lin W.D., Wong M.M., Verslues P.E. (2019). Highly ABA-induced 1 (HAI1)-interacting protein HIN1 and drought acclimation-enhanced splicing efficiency at intron retention sites. Proc. Natl. Acad. Sci. USA.

[95] Castiglioni P., Warner D., Bensen R.J., Anstrom D.C., Harrison J., Stoecker M., Abad M., Kumar G., Salvador S., D’Ordine R. (2008). Bacterial RNA chaperones confer abiotic stress tolerance in plants and improved grain yield in maize under water-limited conditions. Plant Physiol..

[96] Capell T., Bassie L., Christou P. (2004). Modulation of the polyamine biosynthetic pathway in transgenic rice confers tolerance to drought stress. Proc. Natl. Acad. Sci. USA.

[97] Sebastian D., Fernando F.D., Raul D.G., Gabriela G.M. (2020). Overexpression of Arabidopsis aspartic protease *APA1* gene confers drought tolerance. Plant Sci..

[98] Zhang L., Li T., Wang Y., Zhang Y.Y., Dong Y.S. (2019). *FvC5SD* overexpression enhances drought tolerance in soybean by reactive oxygen species scavenging and modulating stress-responsive gene expression. Plant Cell Rep..

[99] Zhao D.Q., Luan Y.T., Shi W.B., Zhang X.Y., Meng J.S., Tao J. (2021). A *Paeonia ostii* caffeoyl-CoA O-methyltransferase confers drought stress tolerance by promoting lignin synthesis and ROS scavenging. Plant Sci..

[100] Vicino P., Carrillo J., Gómez R., Shahinnia F., Tula S., Melzer M., Rutten T., Carrillo N., Hajirezaei M.-R., Lodeyro A.F. (2021). Expression of flavodiiron proteins Flv2-Flv4 in chloroplasts of *Arabidopsis* and tobacco plants provides multiple stress tolerance. Int. J. Mol. Sci..

[101] Guo X.Y., Zhang L., Wang X.Z., Zhang M.H., Xi Y.X., Wang A.Y., Zhu J.B. (2019). Overexpression of *Saussurea involucrata* dehydrin gene *SiDHN* promotes cold and drought tolerance in transgenic tomato plants. PLoS ONE.

[102] Zhang N., Si H.J., Wen G., Du H.H., Liu B.L., Wang D. (2011). Enhanced drought and salinity tolerance in transgenic potato plants with a *BADH* gene from spinach. Plant Biotechnol. Rep..

[103] Goel D., Singh A.K., Yadav V., Babbar S.B., Murata N., Bansal K.C. (2011). Transformation of tomato with a bacterial *codA* gene enhances tolerance to salt and water stresses. J. Plant Physiol..

[104] Duan J.L., Cai W.M. (2012). *OsLEA3-2*, an abiotic stress induced gene of rice plays a key role in salt and drought tolerance. PLoS ONE.

[105] Hema R., Vemanna R.S., Sreeramulu S., Reddy C.P., Senthil-Kumar M., Udayakumar M. (2014). Stable expression of *mtlD* gene imparts multiple stress tolerance in finger millet. PLoS ONE.

[106] Zhu B.C., Su J., Chan M.C., Verma D.P.S., Fan Y.L., Wu R. (1998). Overexpression of a Delta(1)-pyrroline-5-carboxylate synthetase gene and analysis of tolerance to water- and salt-stress in transgenic rice. Plant Sci..

[107] You J., Hu H.H., Xiong L.Z. (2012). An ornithine delta-aminotransferase gene *OsOAT* confers drought and oxidative stress tolerance in rice. Plant Sci..

[108] Nuccio M.L., Wu J., Mowers R., Zhou H.P., Meghji M., Primavesi L.F., Paul M.J., Chen X., Gao Y., Haque E. (2015). Expression of trehalose-6-phosphate phosphatase in maize ears improves yield in well-watered and drought conditions. Nat. Biotechnol..

[109] Park G.G., Park J.J., Yoon J., Yu S.N., An G. (2010). A RING finger E3 ligase gene, *Oryza sativa Delayed Seed Germination 1* (*OsDSG1*), controls seed germination and stress responses in rice. Plant Mol. Biol..

[110] Bae H., Kim S.K., Cho S.K., Kang B.G., Kim W.T. (2011). Overexpression of *OsRDCP1*, a rice RING domain-containing E3 ubiquitin ligase, increased tolerance to drought stress in rice (*Oryza sativa* L.). Plant Sci..

[111] Gao T., Wu Y.R., Zhang Y.Y., Liu L.J., Ning Y.S., Wang D.J., Tong H.N., Chen S.Y., Chu C.C., Xie Q. (2011). *OsSDIR1* overexpression greatly improves drought tolerance in transgenic rice. Plant Mol. Biol..

[112] Qi X.H., Tang X., Liu W.G., Fu X., Luo H.Y., Ghimire S., Zhang N., Si H.J. (2020). A potato RING-finger protein gene *StRFP2* is involved in drought tolerance. Plant Physiol. Biochem..

[113] Wang J.Y., Wang R.T., Mao X.G., Zhang J.L., Liu Y.N., Xie Q., Yang X.Y., Chang X.P., Li C.N., Zhang X.Y. (2020). RING finger ubiquitin E3 ligase gene *TaSDIR1-4A* contributes to determination of grain size in common wheat. J. Exp. Bot..

[114] Chen X., Wang T., Rehman A.U., Wang Y., Qi J., Li Z., Song C., Wang B., Yang S., Gong Z. (2021). Arabidopsis U-box E3 ubiquitin ligase PUB11 negatively regulates drought tolerance by degrading the receptor-like protein kinases LRR1 and KIN7. J. Integr. Plant Biol..

[115] Kim H., Yu S.I., Jung S.H., Lee B.H., Suh M.C. (2019). The F-box protein SAGL1 and ECERIFERUM3 regulate cuticular wax biosynthesis in response to changes in humidity in *Arabidopsis*. Plant Cell.

[116] Ali A., Kim J.K., Jan M., Khan H.A., Khan I.U., Shen M.Z., Park J., Lim C.J., Hussain S., Baek D. (2019). Rheostatic control of ABA signaling through HOS15-mediated OST1 degradation. Mol. Plant.

[117] Wang Q.L., Qu G.P., Kong X.X., Yan Y., Li J.G., Jin J.B. (2018). Arabidopsis small ubiquitin-related modifier protease ASP1 positively regulates abscisic acid signaling during early seedling development. J. Integr. Plant Biol..

[118] Mishra N., Sun L., Zhu X.L., Smith J., Srivastava A.P., Yang X.J., Pehlivan N., Esmaeili N., Luo H., Shen G.X. (2017). Overexpression of the rice SUMO E3 ligase gene OsSIZ1 in cotton enhances drought and heat tolerance, and substantially improves fiber yields in the field under reduced irrigation and rainfed conditions. Plant Cell Physiol..

[119] Wang Y., Ying J.F., Kuzma M., Chalifoux M., Sample A., McArthur C., Uchacz T., Sarvas C., Wan J.X., Dennis D.T. (2005). Molecular tailoring of farnesylation for plant drought tolerance and yield protection. Plant J..

[120] You J., Zong W., Li X.K., Ning J., Hu H.H., Li X.H., Xiao J.H., Xiong L.Z. (2013). The SNAC1-targeted gene *OsSRO1c* modulates stomatal closure and oxidative stress tolerance by regulating hydrogen peroxide in rice. J. Exp. Bot..

[121] Wang F.Z., Wang Q.B., Kwon S.Y., Kwak S.S., Su W.A. (2005). Enhanced drought tolerance of transgenic rice plants expressing a pea manganese superoxide dismutase. J. Plant Physiol..

[122] Bhatt D., Saxena S.C., Jain S., Dobriyal A.K., Majee M., Arora S. (2013). Cloning, expression and functional validation of drought inducible ascorbate peroxidase (*Ec-apx1*) from *Eleusine coracana*. Mol. Biol. Rep..

[123] Wei A.Y., He C.M., Li B., Li N., Zhang J.R. (2011). The pyramid of transgenes *TsVP* and *BetA* effectively enhances the drought tolerance of maize plants. Plant Biotechnol. J..

[124] Lian H.L., Yu X., Ye Q., Ding X.S., Kitagawa Y., Kwak S.S., Su W.A., Tang Z.C. (2004). The role of aquaporin RWC3 in drought avoidance in rice. Plant Cell Physiol..

[125] Xu Y., Jin Z.Q., Xu B.Y., Li J.Y., Li Y.J., Wang X.Y., Wang A.B., Hu W., Huang D.M., Wei Q. (2020). Identification of transcription factors interacting with a 1274 bp promoter of *MaPIP1;1* which confers high-level gene expression and drought stress inducibility in transgenic *Arabidopsis thaliana*. BMC Plant Biol..

[126] Zhang Q., Li J.J., Zhang W.J., Yan S.N., Wang R., Zhao J.F., Li Y.J., Qi Z.G., Sun Z.X., Zhu Z.G. (2012). The putative auxin efflux carrier OsPIN3t is involved in the drought stress response and drought tolerance. Plant J..

[127] Finkina E.I., Melnikova D.N., Bogdanov I.V., Ovchinnikova T.V. (2016). Lipid transfer proteins as components of the plant innate immune system: Structure, functions, and applications. Acta Nat..

[128] Zhu X.Y., Xiong L.Z. (2013). Putative megaenzyme DWA1 plays essential roles in drought resistance by regulating stress-induced wax deposition in rice. Proc. Natl. Acad. Sci. USA.

[129] Islam M.A., Du H., Ning J., Ye H.Y., Xiong L.Z. (2009). Characterization of *Glossy1*-homologous genes in rice involved in leaf wax accumulation and drought resistance. Plant Mol. Biol..

[130] Li L., Li D.L., Liu S.Z., Ma X.L., Dietrich C.R., Hu H.C., Zhang G.S., Liu Z.Y., Zheng J., Wang G.Y. (2013). The maize *glossy13* gene, cloned via BSR-Seq and Seq-Walking encodes a putative ABC transporter required for the normal accumulation of epicuticular waxes. PLoS ONE.

[131] Capell T., Escobar C., Liu H., Burtin D., Lepri O., Christou P. (1998). Over-expression of the oat arginine decarboxylase cDNA in transgenic rice (*Oryza sativa* L.) affects normal development patterns in vitro and results in putrescine accumulation in transgenic plants. Theor. Appl. Genet..

[132] Gupta P.K., Balyan H.S., Sharma S., Kumar R. (2020). Genetics of yield, abiotic stress tolerance and biofortification in wheat (*Triticum aestivum* L.). Theor. Appl. Genet..

[133] Kumar A., Dixit S., Ram T., Yadaw R.B., Mishra K.K., Mandal N.P. (2014). Breeding high-yielding drought-tolerant rice: Genetic variations and conventional and molecular approaches. J. Exp. Bot..

[134] Gautam T., Amardeep, Saripalli G., Rakhi, Kumar A., Gahlaut V., Gadekar D.A., Oak M., Sharma P.K., Balyan H.S. (2021). Introgression of a drought insensitive grain yield QTL for improvement of four Indian bread wheat cultivars using marker assisted breeding without background selection. J. Plant Biochem. Biotechnol..

[135] Merchuk-Ovnat L., Barak V., Fahima T., Ordon F., Lidzbarsky G.A., Krugman T., Saranga Y. (2016). Ancestral QTL alleles from wild emmer wheat improve drought resistance and productivity in modern wheat cultivars. Front. Plant Sci..

[136] Wang N., Cheng M., Chen Y., Liu B.J., Wang X.N., Li G.J., Zhou Y.H., Luo P., Xi Z.Y., Yong H.J. (2021). Natural variations in the non-coding region of *ZmNAC080308* contributes maintaining grain yield under drought stress in maize. BMC Plant Biol..

[137] Guo T.T., Yu X.Q., Li X.R., Zhang H.Z., Zhu C.S., Flint-Garcia S., McMullen M.D., Holland J.B., Szalma S.J., Wisser R.J. (2019). Optimal designs for genomic selection in hybrid crops. Mol. Plant.

[138] Wang B.B., Lin Z.C., Li X., Zhao Y.P., Zhao B.B., Wu G.X., Ma X.J., Wang H., Xie Y.R., Li Q.Q. (2020). Genome-wide selection and genetic improvement during modern maize breeding. Nat. Genet..

[139] Cui Y.R., Li R.D., Li G.W., Zhang F., Zhu T.T., Zhang Q.F., Ali J., Li Z.K., Xu S.Z. (2020). Hybrid breeding of rice via genomic selection. Plant Biotechnol. J..

[140] Rembe M., Zhao Y.S., Jiang Y., Reif J.C. (2019). Reciprocal recurrent genomic selection: An attractive tool to leverage hybrid wheat breeding. Theor. Appl. Genet..

[141] Hanna R.E., Doench J.G. (2020). Design and analysis of CRISPR-Cas experiments. Nat. Biotechnol..

[142] Chen K.L., Wang Y.P., Zhang R., Zhang H.W., Gao C.X. (2019). CRISPR/Cas genome editing and precision plant breeding in agriculture. Annu. Rev. Plant Biol..

[143] Vaidya A.S., Helander J.D.M., Peterson F.C., Elzinga D., Dejonghe W., Kaundal A., Park S.Y., Xing Z.N., Mega R., Takeuchi J. (2019). Dynamic control of plant water use using designed ABA receptor agonists. Science.

[144] Cao M.J., Zhang Y.L., Liu X., Huang H., Zhou X.E., Wang W.L., Zeng A., Zhao C.Z., Si T., Du J.M. (2017). Combining chemical and genetic approaches to increase drought resistance in plants. Nat. Commun..

[145] Park S.Y., Peterson F.C., Mosquna A., Yao J., Volkman B.F., Cutler S.R. (2015). Agrochemical control of plant water use using engineered abscisic acid receptors. Nature.

[146] Lavarenne J., Guyomarc’h S., Sallaud C., Gantet P., Lucas M. (2018). The spring of systems biology-driven breeding. Trends Plant Sci..

[147] Liu T., Fedak G., Zhang L.Q., Zhou R.R., Chi D., Fetch T., Hiebert C., Chen W.J., Liu B.L., Liu D.C. (2020). Molecular marker based design for breeding wheat lines with multiple resistance and superior quality. Plant Dis..

[148] Yu Y., Qian Q. (2019). Rice breeding: A long noncoding locus with great potential. Mol. Plant.

[149] Rai G.K., Rai N.P., Rathaur S., Kumar S., Singh M. (2013). Expression of *rd29A*::*AtDREB1A*/*CBF3* in tomato alleviates drought-induced oxidative stress by regulating key enzymatic and non-enzymatic antioxidants. Plant Physiol. Biochem..

